# The Odontoblasts: Their Formation, Function and Power of Appropriation

**Published:** 1890-10

**Authors:** W. N. Murphy

**Affiliations:** La Grange, Texas.


ARTICLE III.

### THE ODONTOBLASTS: THEIR FORMATION, FUNCTION AND POWER OF APPROPRIATION.

BY W. N. MURPHY, D.D.S., LA GRANGE, TEXAS.

It may be that the study of Hystology is tiresome and very unsatisfactory to a great many, and it has been said that in certain societies when a paper is to be read upon this subject that many leave the room. Now if there be such a man in this association I wish to disabuse your mind. When you neglect Hystology you neglect the word of life to your profession, it is the keynote to your success, and until you base your treatment on it you can not hope for specific results, and the man who guesses at results is but to find himself sooner or later bound fast in the clutches of Pathology. But as the whole field of Hystology embraces too much for one paper it will be impossible for me to treat of the various stages of even the first or second dentition; indeed in our daily practice it becomes a matter of little consequence to us to know just what day or week of embryonal life the enamel organ begins its formation; we do not care to know what day the dental falical is served. it is of but little significance to us to know just when the foetal jaw begins to ossify, but when we come to consider that which maintains, nourishes, and supports the tooth throughout its natural life, it becomes a matter of profound interest as well as the most practical value to us in our treatment for dental caries to fully understand and appreciate this method of constructive metamorphosis, and in calling your attention to the odontoblasts, their formation, and function, I trust you will follow me closely so that if I run too far astray you may bring me back. It is true that we are indebted to but a limited number of the profession for the progress that we have already made in this special branch of dentistry, which has been handed down to us for the past decades, and it may appear to some that we have long since reached the zenith in this particular branch, but when we come to look around, and view our many failures, when we see the many changes that are being made by the revolution of time and the rapid strides that are being made in other scientific researches, especially other departments of dentistry, we are led to the conclusion that there is still room for further advancement in the study of dental as well as general Hystology, hence I make this apology for presenting what may be a direct antagonism to what has been said heretofore. But now to the point; these cells, like all other cells, have a special function to perform peculiar to the organ to which they belong or represent. Upon and near the surface of the dental papilla may be seen these cells in all of the various stages of formation, from the fully developed cell back to those just beginning their formation in the deeper layers of the connective tissue, new cells being formed all the time, and all the way on down from the earlier formation of the dental papilla to the time the tooth has been erupted; yes, further on until it has completed its natural function, or the entire pulp chamber has been completely filled with consecutive layers of physiological dentine, and I hope you will note the full significance of the word physiological in this connection, for I fear there is not enough distinction made between physiological and secondary dentine, but as it does not come under the province of this paper to treat on the formation of secondary dentine I shall pass on to my subject. In the first stages of the development of these cells we find deeper down in the connective tissue of the papilla small nuclei or protoplasmic masses sending out toward the surface of the papilla a ganglion or process which we may justly call the dental fibril, (of which we shall make mention later on) and which spread out into and through the membranaeboris anastomosing with the fibrils of the older cells. As the cells are completed they assume more of an oblong shape, sending back into the deeper tissue a ganglious or process just as they do in the first place, sending them to the surface. Upon these backward processes or ganglii are started and formed new cells to take the place of the old ones, as they have completed their function and passed away or been made into layers of dentine. They are each one dependent upon the other in that each cell anastomoses with another by this ganglion heretofore mentioned, were this not the case the chain of dental fibrils would be broken as soon as the first layers of dentine were completed, and the new cells begin their individual labor, but by this gangliatic connection and this dependence of the new cells upon the old, and the old upon the new, we can see how the new cells begin their work just where the old ones leave off and thereby keep up the fibril connection between the last formed cells and the bond of union between the enamel and dentine. Without this connecting link in the fibrils the outer layers of dentine and enamel would cease to have any connection whatever with the newer layers of dentine, and they would separate from each other as the bark from a tree. The dentine of its self has no power of conductivity and without this fibril connection there could be no response to thermal changes, there would not be that accute sensitiveness that we find at the union of enamel and dentine where the fibrils anastomose so freely, but last but not least the enamel and outer layers of dentine would fail to receive the food necessary for their maintainance, and we would soon see that disintegration, or drying out of the waters of crystalization that we so often see in teeth with devitalized pulp, where there seems to be a tendency for the dentine and enamel to pull away from each other. Dry the sap out of a green tree and see how readily the bark will hull off of it.

Pardon me for referring to that renowned Hystologist and Microscopist Dr. Andrews, to whom I acknowledge my indebtedness although his opinions as given in his able and exhaustive article, and published in Cosmos of 1888, page 221, are in direct antagonism to the line of thought presented in this paper. According to his opinion one would be lead to believe that there were but one set of odontoblastic cells, and that certain fibered cells play a part in dentine formation, which we hold is erroneous. It is immaterial what name we give to designate the cells if they are all intended to do the same kind of work, which we claim they do, but as the term odontoblast has become so commonly used and I think properly, so I shall continue to designate them all as odontoblasts and endeavor to prove that they are all the same. According to his idea, as soon as the adontoblasts (which must be about the size of a grain of wheat) have finished their work the power of Dentine must be at an end, but assuming this to be true, (which we do not), and giving the odontoblasts about ½500 of an inch in length we can see that the amount of dentine produced would be inadequate to the demand. In fact, it would be a mockery of dentine; and granting on the other hand that the cells and membranaeboris have the power; (as claimed by Dr. Jewell, see Cosmos, August, 1886), of retreating as the walls of dentine which they are throwing up, begin to encroach upon them in order to keep up their work, and thereby produce an adequate supply of dentine, and at the same time keep up the fibril connection. What would be the natural sequence? We would soon have jungled up in the remains of the pulp chamber a bundle of cells to the exclusion of the very material with which they are to build dentine, but this cannot be true, it is contrary to the laws of constructive metamorphosis. But the cells being under the direct control and management of the nervous system, each one being supplied by one or more nerve fibers of that branch of the 5th pair of nerves which supply the Papilla by breaking up into many minute fibres and permeating the entire pulp and dentine, their power of appropriation being increased by what ever will stimulate these nerve terminations. The cells under the management of the nervous terminations have the power of taking up the material conveyed to them through circulation and producing calcosphorites beginning at the periphery and proceeding inward at the expense of the size of the pulp, each layer making the pulp smaller. But the question may be asked just here, do the cells diminish in size to correspond to the diminution of the size of the pulp. Yes, they diminish in size until they become a part of the fibril, loosing their identity as a cell. The tubular dentine coming on down to the end of the cell, and by reason of its semi-calcification has the power of squeezing in on the end of the cells so as to gradually reduce them in size to only an enlarged portion of the fibril, (which probably give rise to the opinion that certain fibril cells play a part in dentine formation) and just here the newly formed cells begin their work forming dentine, and as the new cells are formed they diminish in number, one cell connecting with two or more old cells, by the backward prolongations of the several old cells running into the new one, or the new one rather being formed as it were just where the two run together. The new cells being as large and active in the later stages of dentine formation as in the earlier or first set of cells.

Now having gone briefly over the formation and function of these cells, we come to the consideration of another very important point, and one I fear has never received the thought that it really demands, viz: The power of these cells to appropriate the food necessary for building up the several types of teeth found in different individuals.

Are all teeth endowed with the same functional activity? Are they all possessed with the same density of structure? Do they all have the same number of odontoblastic cells? No!

Why such a variance in functional activity? Why such a descrepancy in the density of the teeth of two or more individuals?

Perhaps some of the phosphate feeders will tell us that it is due to an insufficient quantity of the different inorganics that go to build up the asseous structure, but the writer will tell you that it is due to a want of appropriation of these salts caused by a low power of nervous force and an insufficient number of odontoblasts to perform this their peculiar function.

We have all seen fair shells of enamel filled with the poorest quality of dentine.

In the soft, white chalky teeth, upon which we usually find the largest amount of salivary deposit and that of the softest variety, we usually find the smallest number of odontoblastic cells, while in the hard, dense variety upon which we usually find the smallest quantity of deposit, and it is of the hardest variety, (except in persons past the meridian of life), we find the greatest number of odontoblastic cells the increased number of cells being able to appropriate a large percentage of the salts necessarily making precipitation of inorganic in the saliva very slow and in very small quantities at any one time, just in the same ratio that the cells vary in number will the teeth vary in density, also the calculous vary in quantity and density.

But now to the point of appropriation. The blood being the means of transportation it is the vehicle that carries the food throughout the entire economy, necessary for constructive metamorphosis and we must therefore stop to consider whether or not it confines its work to such articles of food as are found in the animal and vegetable kingdom or whether it takes up and appropriates such combinations as may be roughly prepared by our hands. We know that it only has the power of taking up such articles as can be held in solution and conveyed to these remote parts. Yet we grant that she can and does take up and carry into every capillary that which is foreign to her natural demands and which do not promote constructive metamorphosis, but rather have the power of dragging out that which impedes nature in her work, we have a striking example of this in the results following a mighty dose of calomel or sulphate of magnesia but have we any proof that our earthy mixtures are ever appropriated and converted into that which protects the pulp from that which is now properly supposed to be one of the prime factors in dental caries viz: the "Bug."

Has any chemist ever demonstrated that a given quantity of carbonate of lime, carbonate of magnesia, etc., will give the same chemical compound or in the same form as that found in the animal and vegetable kingdom?

Then if all this be true we must give our attention to our food and see whether or not it contains a sufficient quantity and whether or not the cells are able to appropriate it or not.

When the physician gives drugs for the purpose of conveying oxygen will he give it in the form of a paper of tacks or will he give it in such form as will be most likely to be readily assimilated? But when he gives ten grains Fe Sulphate to his patient daily does it not seem reasonable that he could get more than forty-five grains from the human body at any one time? I suppose he could if they had the power of appropriating all that doctors could stuff down them. Then in the face of all these facts can we crowd upon these few cells, this little band of operatives in this dentine manufacturing establishment phosphates in different forms and in such quantities as will build up large and strong teeth, or will we overwork them by sending them more material than they can spin up and work into cloth of dentine, which must be sent back to the salivary and other glands to be gotten rid of or thrown out of the way as waste? The different glands unquestionably have the power of extracting the excess of inorganics from the blood and throwing them out as waste as is evidenced by the analysis of normal submaxilary saliva as per Biddle and Schmidt, which is as follows:

| | |
|---|---|
| Water | 991.45 |
| Organic Matter | 2.29 |
| Sodium Chloride / Calcium Chloride | 4.50 |
| Calcium Carbonate | |
| Calcium Phosphate | |
| Magnesium " | 1.16 |
| | 1.000 |

We also have a further example of this in the solids found in normal urine and the perspiration from our bodies as given by Martin and not necessary to recapitulate. But some might say that the solids which we find in normal saliva are intended to play a part in digestion but when we find that the saliva only acts on starchy food and this being done by the active principal ptylin it knocks the prop from under any such claims, besides it would be unreasonable, and not expect that nature has to resort to such a round-about and unnatural way of digesting the same thing in food so that after a careful consideration of all the premises we are forced up to the conclusions that it is not safe to expect to build up a better class of teeth for the individual, as nature has provided a way for refusing or throwing out all that she can not use.

Martin's analysis of one day's food for an adult is sodium chloride 482.9 and other salts in various proportion, so that we can see that in the course of one year we would take in the form of food more inorganics than the entire weight of all the bones and teeth of our bodies, and as it has never been claimed or proven that man is entirely reproduced in the short space of one year it is hardly reasonable to presume that this vast amount is all appropriated, else there would be no limit to the growth of man, and until some man can show me some more reasonable theory I shall hold that the low order of teeth is due to a low power of nervous force and an insufficient number of odontoblastic cells to appropriate a sufficient quantity of inorganics to build them up to the highest standard.—*Texas Dental Journal.*

